# Microbial Response to Micrometer-Scale Multiaxial Wrinkled Surfaces

**DOI:** 10.1021/acsami.2c08768

**Published:** 2022-06-14

**Authors:** Luca Pellegrino, Lukas Simon Kriem, Eric S. J. Robles, João T. Cabral

**Affiliations:** † Department of Chemical Engineering, Imperial College London, London SW7 2AZ, United Kingdom; ‡ Fraunhofer Institute for Interfacial Engineering and Biotechnology IGB, Nobelstrasse 12, 70569 Stuttgart, Germany; § Procter & Gamble, Newcastle Innovation Centre, Newcastle upon Tyne NE12 9TS, United Kingdom

**Keywords:** surface topography, roughness, antimicrobial, antibacterial, PDMS, patterning, wrinkling

## Abstract

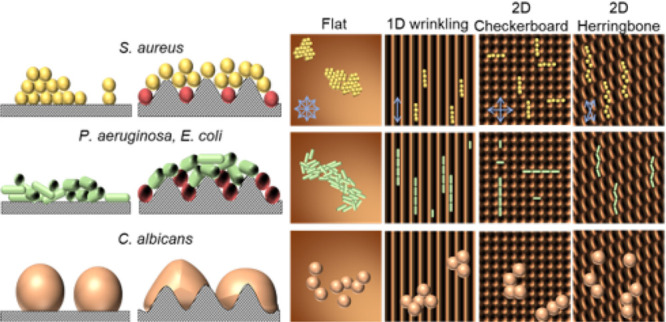

We investigate the effect of micrometer-scale surface wrinkling on the attachment and proliferation of model bacteria (*Staphylococcus aureus*, *Pseudomonas aeruginosa*, and *Escherichia coli* K12) and fungi (*Candida albicans*). Specifically, sinusoidal (1D), checkerboard (C), and herringbone (H) patterns were fabricated by mechanical wrinkling of plasma-oxidized polydimethylsiloxane (PDMS) bilayers and contrasted with flat (F) surfaces. Microbial deformation and orientation were found to correlate with the aspect ratio and commensurably with surface pattern dimensions and local pattern order. Significantly, the proliferation of *P. aeruginosa* could be described by a linear scaling between bacterial area coverage and available surface area, defined as a fraction of the line integral along each profile with negative curvature. However, in the early stages of proliferation (up to 6 h examined), that C and H patterns disrupt the spatial arrangement of bacteria, impeding proliferation for several hours and reducing it (by ∼50%) thereafter. Our findings suggest a simple framework to rationalize the impact of micrometer-scale topography on microbial action and demonstrate that multiaxial patterning order provides an effective strategy to delay and frustrate the early stages of bacterial proliferation.

## Introduction

Microbial colonization of biotic and abiotic surfaces is a complex and multistage process that involves locating, approaching and sensing the proximity of the surface.^1,2^ Interaction with solid surfaces involves cascades of chemical gradients, or chemotaxis, and specifically “quorum sensing” (QS), in which bacterial cells exchange small extracellular molecules to coordinate the formation of microbial communities.^3−5^ In addition to chemical sensing, bacteria are able to sense surface mechanics through their appendages and constrained movements, in order to perceive the presence of other attached cells.^6^

Surface colonization poses significant challenges in wide-ranging contexts, including biomedical implantable devices, as the formation of microbial communities or biofilms can result in infection, implant failure, and revision surgeries.^7−10^ Such device-related infections are difficult to treat as a biofilm envelopes the bacteria, protecting them from the immune response and antibacterial treatments.

Substrate topography impacts many cellular developmental processes, imparting the ability of cells to orient themselves, migrate, and produce organized cytoskeletal arrangements in response to dynamic changes in their physical microenvironments.^11,12^ Shape adaptation leverages the intrinsic anisotropy in the mechanical properties of extracellular matrices (ECMs), important in complex cell dynamics mechanisms known as “contact guidance”^13−17^ In quiescent conditions, i.e., in the absence of flow, hydrostatic forces, and gravity within the liquid medium, govern the initial displacement of bacteria, followed by electrostatic and chemical adhesion forces, including those mediated by pili, flagella, and adhesins that govern attachment.^18^ By contrast, under dynamic conditions investigated with prescribed flow cells, microtopographies have been found to create recirculation zones that disrupt the otherwise uniform velocity distributions characteristic of planar surfaces.^19^

Nano- and microscale topography has been shown to impact bacterial attachment and subsequent biofilm formation through a range of mechanisms.^20−25^ Nanoscale patterns can affect the surface physicochemical forces and free energy, cell membrane deformation, and chemical gradients at the solid–liquid interface, mimicking the contact-killing, biocidal behavior of natural occurring nanopatterns such as the epicuticular structures of cicada and dragonfly wings.^26−29^ Microactive mechanisms, on the other hand, affect surface hydrodynamics, surface entrapment, microbial ordering and segregation, and surface conditioning.^6,21,30−32^ Surface topographies commensurate with microbial dimensions (on a micrometer scale) are able to direct spreading and proliferation of different microbial strains on varying materials. The spatial periodicity, amplitude, profile shape, and distribution of topographic patterns (including tortuosity) are all expected to play an important role in bacterial attachment. For example, topography-mediated antifouling effects were investigated on line patterns,^33−36^ micropits, or wells of (circular or honeycomb),^21,31,37,38^ square,^39−41^ and hexagonal lattices,^42,43^ suggesting that bacteria are constrained to prescribed arrangements that reduce the area fraction available for fouling,^31,44^ the number of contact points for adhesion,^45,46^ and surface curvature,^47^ thus reducing bacterial motility and interconnections.

Studies of topographical effects in microbial fouling generally use some (scalar) “roughness” metric to describe surfaces, which has become prevalent in literature.^48−50^ Expectedly, this has led to inconsistency of results and contradictions given the multiple definitions of roughness^51^ and measurement technicalities, but primarily due to the fact that distinct surface topographies can yield identical “roughness” descriptors.

**Figure 1:**
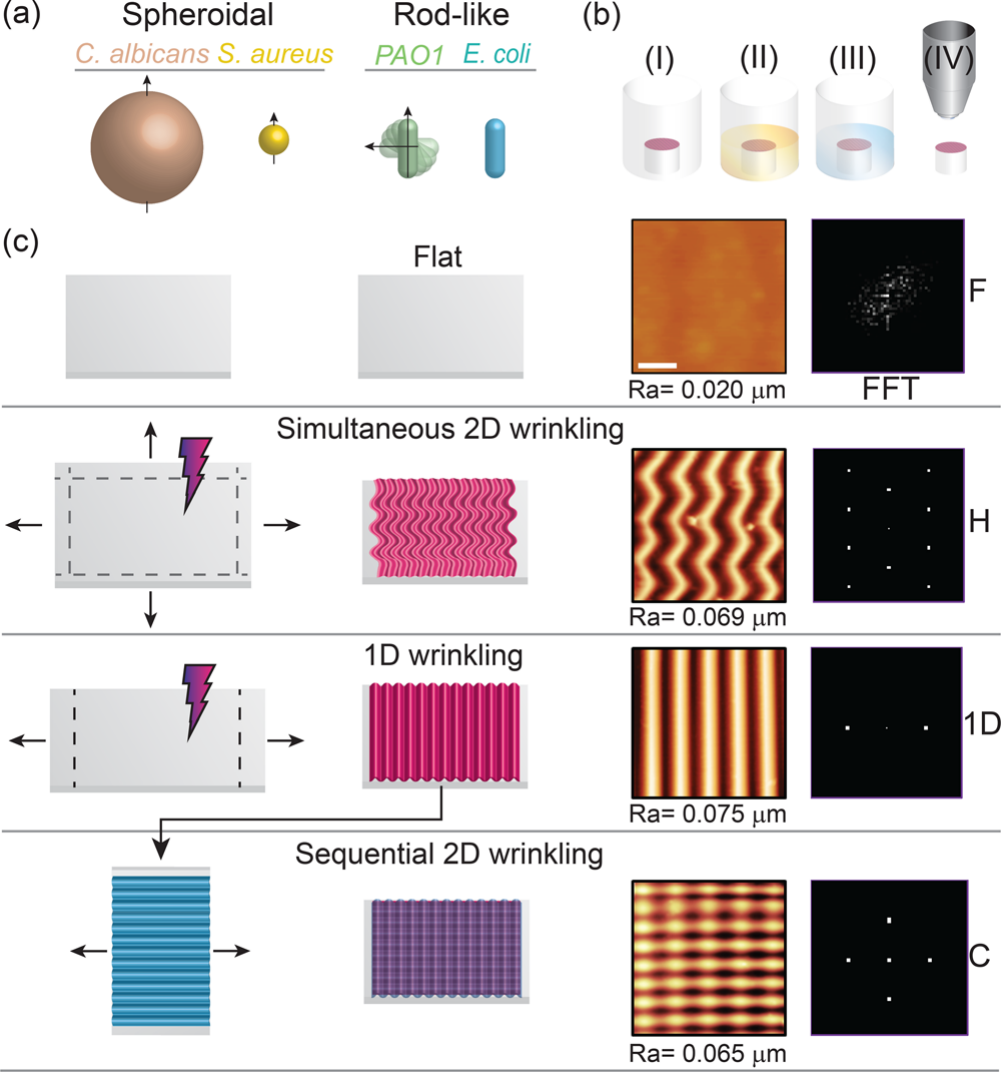
Microorganism culture and surface fabrication protocols. (a) Schematic of the three bacterial strains and fungus employed in the experiments: Gram-negative rodlike *P. aeruginosa* (PA01) and *E. coli* K12, and spheroidal Gram-positive *S.aureus*, and fungus *C. albicans*. (b) (I) PDMS substrates (5 mm circular) were attached to the bottom of polystyrene well plates. (II) Microbial inoculum was deposited onto the PDMS coupons. (III) At prescribed times, substrates were dip-washed, dried, and (IV) imaged. (c) PDMS surface topographies and corresponding AFM images (scale bar 2 μm): flat, reference substrate (F); herringbone pattern (H) fabricated by biaxial strain; sinusoidal wrinkled surface (1D) fabricated by uniaxial strain; and checkerboard pattern (C) fabricated by sequential, orthogonal wrinkling. Surfaces 1D, H, and C were designed to exhibit similar surface roughness (reported as *R*_a_, arithmetical roughness) but display different in-plane order, characterized by the fast Fourier transforms (FFT) shown on the right.

In this work, we examine the impact of distinct topographies, with approximately the same “roughness”, on microbial response and proliferation, illustrated in Figure 1. We comparatively examine a reference flat (F) surface, with sinusoidal (1D), checkerboard (C), and herringbone (H) patterns fabricated by the surface wrinkling of polydimethylsiloxane bilayers,^52,53^ exhibiting a wavelength of λ ≃ 2 μm, an amplitude of *A* ≃ 0.2 μm (and thus aspect ratio of *A*/λ ≃ 0.1), and a roughness of *R*_a_ ≃ 0.07 μm and *R*_q_ ≃ 0.08 μm (detailed below). We select three bacterial strains: spheroidal *S. aureus* and rodlike *P. aeruginosa* and *E. coli* K12, of dimensions commensurate with the micrometer-scale pattern wavelength; for comparison, we include a spheroidal fungus *C. albicans*, several times larger than the pattern features, illustrated in Figure 1a. By employing imaging and microbial culture methods (Figure 1b), we seek to isolate possible effects of surface topography and tortuosity Figure 1c) on microbial attachment and proliferation.

## Experimental Section

### Surface Patterning

Polydimethylsiloxane (PDMS, Sylgard 184, Dow Corning) was prepared at a 10:1 prepolymer/cross-linker ratio by mass, stirred, degassed under vacuum, deposited onto a glass plate, cured at 75 °C in a convection oven for 1 h, and cut into coupons of length, width, and thickness of 3 cm × 2.5 cm × 2.5 mm. PDMS has approximate surface energy of 18–20 mN/m. Wrinkled surfaces were fabricated by plasma oxidation under strain, followed by strain release and replication onto fresh PDMS (to eliminate the hydrophilic effects of plasma exposure and gradual hydrophobic recovery), as illustrated in Figure 1c.

Sinusoidal patterns (1D) were fabricated by uniaxial strain of a PDMS coupon, using a strain stage controlled by a linear actuator and imposing the required prestrain ϵ = (*L*_1_ – *L*_0_)/*L*_0_ at a speed of 0.02 mm/s with a precision of ±0.001 mm; *L*_0_ and *L*_1_ are, respectively, the initial and final distances between the clamps. Surface plasma oxidation was carried out using a 40 kHz Diener Plasma (Femto), operated at power *p* = 20 W, oxygen gas (BOC, 99.5%) pressure *P* = 1 mbar (monitored by pressure sensor TM 101, Thermovac), for a duration τ = 120 s. A prestrain ϵ = 20% was imposed during oxidation and was released at a controlled speed of 0.2 mm/s (to minimize surface cracking), resulting in 1D sinusoidal wrinkles, with periodicity λ and amplitude *A*, determined by *p*, *P*, τ, and ϵ_prestrain_, as previously reported.^54,55^

Herringbone (H), or chevron, patterns^56^ were formed by simultaneous 2D wrinkling, whereby a PDMS slab of 5 × 5 cm^2^ is equi-biaxially stretched (ϵ_*x*_ = ϵ_*y*_ = 10%) and plasma-oxidized at *p* = 20 W for τ = 120 s, followed by strain release.

Symmetric checkerboard (C), or “egg-tray”, patterns were fabricated by a sequential 2D wrinkling procedure, reported previously.^57,58^ A first generation of 1D wrinkles (master) is obtained at *p* = 20 W and τ = 120 s and then replicated onto a fresh PDMS coupon in order to yield a fresh, stress-free PDMS surface, eliminating the brittle glassy layer resulting from the plasma oxidation step. The replica is fabricated by casting liquid PDMS onto the 1D master, previously coated with octadecyl trichlorosilane (ODS, Acros Organics, 95%) by adsorption from the vapor phase in a desiccator for 30 min, and cross-linking at 75 °C for 1 h, before peeled off from the master. The replica is then uniaxially stretched in the perpendicular direction with respect to the first generation and plasma treated, using the same *p* and τ, to obtain once releasing the prestrain, a secondary wrinkling generation. The orthogonal wave superposition of the two wrinkling generations yields a symmetric checkerboard with appropriate prestrains ϵ_1,2_.

### Pattern Characterization

Surface topographies were characterized using atomic force microscopy (AFM) using a Bruker Innova microscope, in tapping mode at 0.2 Hz, equipped with Al-coated Si tips (MPP-11100-W, Bruker). AFM data analysis was carried out using the Gwyddion software, averaging wavelengths and amplitudes over a series of three measurements recorded over different 100 μm × 100 μm scanned images; λ and *A* were computed from peak-to-peak distances and half of the peak-to-valley heights, respectively. The surface roughness is normally reported in terms of the arithmetical mean deviation of the profile, , or alternatively as the root-mean-square, . For the surfaces investigated, we sought to keep the pattern roughness constant and obtain *R*_a_(1D) = 0.075 μm and *R*_q_(1D) = 0.090 μm; *R*_a_(H) = 0.069 μm and *R*_q_(H) = 0.085 μm; *R*_a_(C) = 0.065 μm and *R*_q_(C) = 0.080 μm; *R*_a_(F) = 0.020 μm and *R*_q_(F) = 0.030 μm, with an uncertainty of approximately δ*R* ≈ 0.005 μm. Water contact angle measurements (Figure S9) reveal that the 2D samples exhibit a slightly lower contact angle, namely 85° for C and 98° for H surfaces, than 1D (105°) and flat F (106°) surfaces. Since the patterned (plasma-exposed) samples were replicated into virgin PDMS, these (modest) changes are ascribed solely to pattern topography since the surface chemistry is identical (Figure S1).

### Bacterial Strains and Inoculation

Three bacterial and one fungal strains were employed in the experiments: bacteria, Gram-positive *Staphylococcus aureus* (DSM346) and two Gram-negative *Pseudomonas aeruginosa* (PAO1, DSM19880) and *Escherichia coli* K12 (DSM498), and fungus *Candida albicans* (ATCC10231). The microorganisms were first imaged via scanning electron microscopy (SEM) to characterize their shape and dimensions, using a LEO Gemini 1525 FEGSEM microscope (gold sputtering was employed to reduce surface charging).

Precultures were started by transferring the strains in tryptic soy broth (TSB, Merck 1.05459.0500) medium in aerobic conditions at 32.5 °C. After 72 h, the strains are transferred into a broth medium constituted by 5 mL of TSB medium + 50 μL of bacteria medium from the initial culture and incubated overnight under aerobic conditions at 32.5 °C. Serial dilution of liquid culture and plating on TSB plates was carried out to determine colony forming units (CFU) (typically ∼10^7^), followed by incubation for 24 h at 32.5 °C, allowing an uniform incubation of samples with the same amount of CFUs.

The wrinkled substrates were cut into cylindrical shapes of 0.5 mm diameter, placed in a 48-well microtiter plates (VWR) with the surface facing up, and inoculated with 1000 μL of liquid culture under aerobic conditions at 32.5 ^◦^C, as depicted in Figure 1b. After each time point at 2, 4, and 6 h, three samples from each surface were removed and rinsed in DI water. In order to minimize potential impact of sample processing on alignment, rinsing was carried out by slow, vertical dipping of the samples, at a speed of ∼0.03 mm/min, to eliminate (or greatly minimize) shear effects. The relative orientation of 1D patterns with respect to dipping was also varied with no discernible effect. To remove excess water, the samples were dried for 15 min at 32.5 °C.

### Sample Preparation for SEM/AFM Imaging

Prior to AFM and SEM imaging, microorganisms attached to the wrinkled structures were dehydrated and immobilized. Each sample was put into a new well of a 48-well plate containing 1000 uL of 2% glutardialdehyde at room temperature for 60 min. Glutardialdehyde was removed and replaced by 25% *v*/*v* technical-grade ethanol (Sigma-Aldrich, CAS No. 64–17–5) and incubated at room temperature for 15 min. This dehydration step was repeated for an additional three times at 50, 75, and 96% *v*/*v*, respectively. After the last dehydration step, the samples were air-dried at room temperature. Surface characterization was carried out using contact mode AFM (Bruker Innova microscope) at 0.1 Hz with Au-coated Si_3_N_4_ tips (MLCT), acquiring micrographs at different windows of 20 × 20, 50 × 50, and 100 × 100 μm^2^. Microbial distribution and orientation maps were computed using ImageJ software.

## Results and Discussion

### Microbial Attachment on Commensurate Wrinkled Topographies

**Figure 2:**
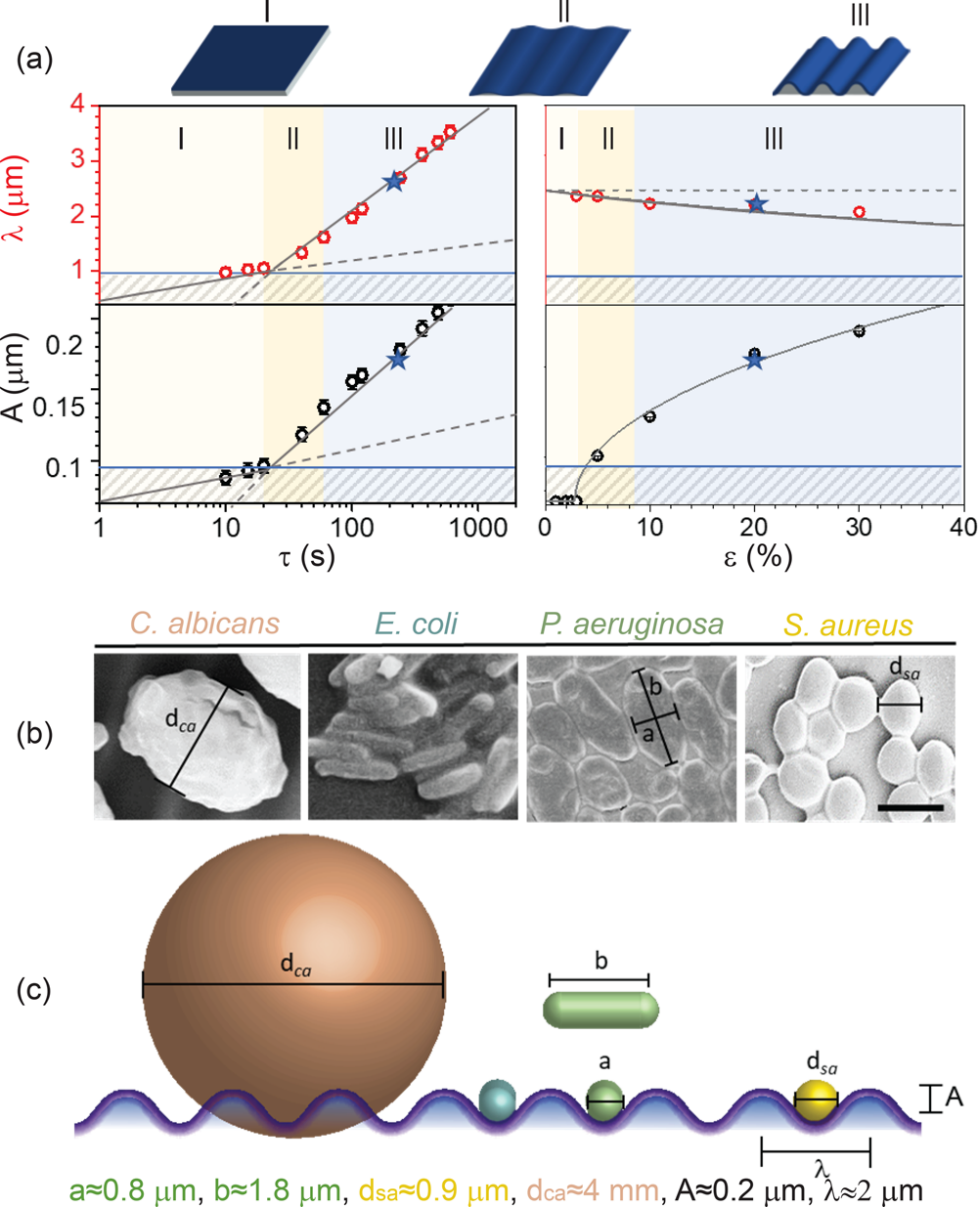
(a) Wrinkling wavelength and amplitude as a function of plasma exposure time τ (left) and mechanical prestrain ϵ (right), employing a 40 kHz oxygen plasma, operating at *p* = 20 W and *P* = 1 mbar. The three sections represent the (I) planar, (II) induction and (III) propagation regimes.^54,55^ Blue stars indicate λ ≈ 2 μm and *A* ≈ 0.2 μm, corresponding respectively to τ = 200 s and ϵ = 20% chosen for the experiments. (b) SEM micrographs of the microorganisms. Black bars indicates how the characteristic dimensions reported in Table. 1 have been measured. (c) Microorganism scales compared to a 1D wrinkling pattern (λ ≈ 2 μm, *A* ≈ 0.2 μm). Microorganisms are color-coded according to Figure 1.

The surface pattern dimensions were designed (Figure 2a) to be commensurate with the bacterial dimensions, and smaller than those of the fungus, as shown in the SEM images in Figure 2b and the cross-sectional schematic of Figure 2c. In simple terms, our microbial model systems are classified in terms of their cross-sectional dimension *a*, length *b*, aspect ratio *b*/*a*, and Gram stain status, as summarized in Table 1. With *a* ≈ *b*, and thus *b*/*a* ≈ 1, *S. aureus* and *C. albicans* are “spheroidal”. With *b* > *a* and *b*/*a* > 1, *P. aeruginosa* and *E. coli* K12 are termed ellipsoidal, or “rodlike” (exhibiting similar cross section and varying aspect ratio, in order to examine in detail the role of topography).

**Table 1:** Microbial Cross-Section *a*, Length *b*, and Aspect Ratio *b*/*a* Estimated by SEM

	Gram stain	*a* (μm)	*b* (μm)	aspect ratio (*b*/*a*)
*S. aureus*	+	0.8 ± 0.3	0.9 ± 0.3	≈1.1
*P. aeruginosa*	–	0.7 ± 0.2	1.7 ± 0.2	≈2.4
*E. coli* K12	–	0.6 ± 0.2	1.9 ± 0.2	≈3.2
*C. albicans*	+ (yeast)	3.8 ± 1.5	4.1 ± 1.5	≈1.1

The surface wrinkling pattern length scales were tuned by the plasma oxidation and mechanical strain parameters, following our previous work.^54,55,57−59^ The glassy skin thickness *h* induced by oxygen plasma exposure of PDMS evolves through a frontal propagation process and can be tuned by plasma power, oxygen pressure, and exposure time.^54,55^ In the low deformation limit, the wavelength λ and amplitude *A* of a wrinkled bilayer under strain is given by^60^
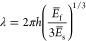
1
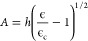
2where *h* is the film thickness, *E̅*_f_ and *E̅*_s_ are, respectively, are the in-plane strain moduli of the film and substrate, given by *E̅* = *E*/(1 – ν^2^), where *E* is the Young’s modulus, ν is the Poisson ratio (≃ 0.5 for PDMS), and ϵ_c_ is the critical strain
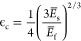
3that must be exceeded to trigger the wrinkling instability. For this study, we have selected λ ≃ 2 μm and *A* ≃ 0.2 μm, as shown by the star (★) symbols in Figure 2a, yielding undulating profiles commensurate with bacterial cell dimensions and therefore potentially able to interfere their initial attachment. The fungus *C. albicans* was chosen as a model for larger dimensions (∼2–3λ). For comparison, a schematic of microbial size and sinusoidal pattern is shown in Figure 2c. In short, our experimental design is as follows: We examine the impact of distinct surfaces of *constant* roughness (three patterns and planar reference), on the initial attachment and proliferation of (4) microbial strains, measured over time (0, 2, 4, and 6 h); each condition is measured in triplicate (thus 4 × 4 × 4 × 3 = 192 individual samples).

**Figure 3:**
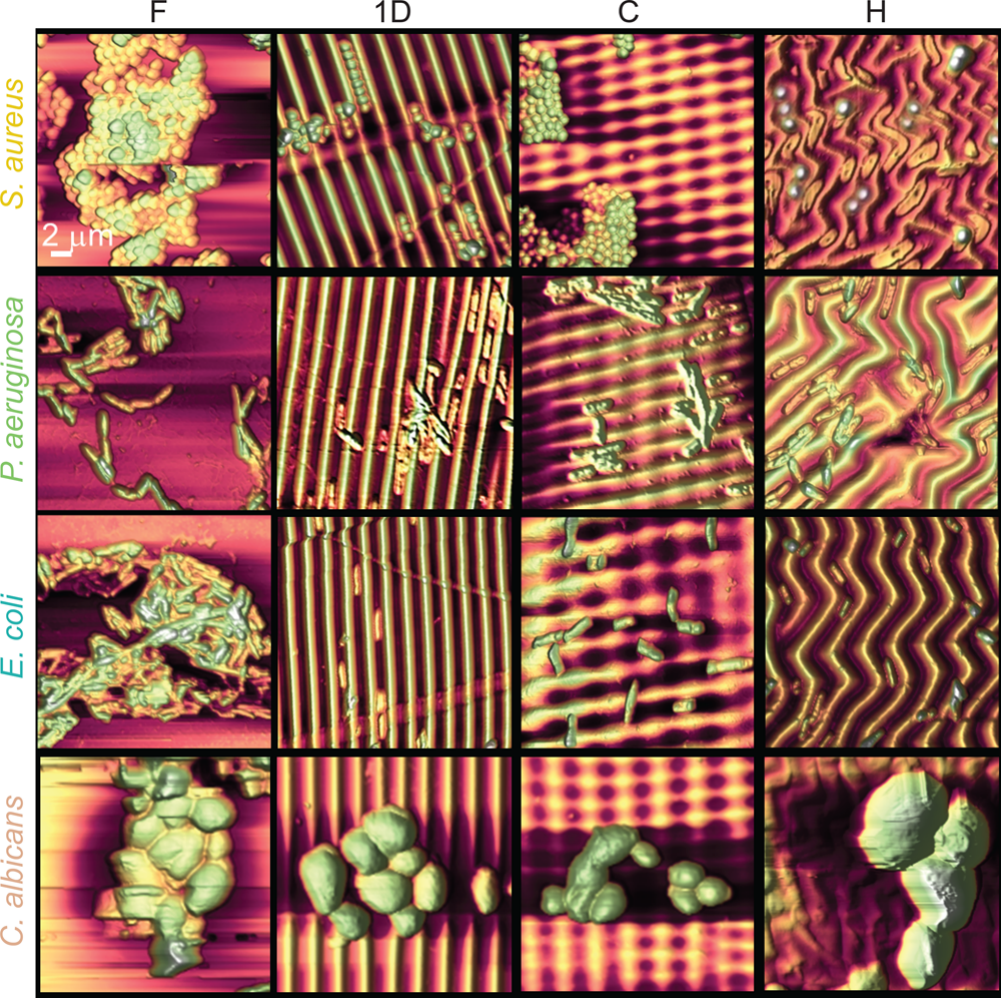
AFM images (20 × 20 μm^2^) of bacteria *S.aureus*, *P. aeruginosa*, and *E. coli* K12 and fungus *C. albicans* on the four surface patterns after 6 h incubation time: F: flat, 1D: uniaxial sinusoidal, C: checkerboard, and H: herringbone. Bacteria are randomly oriented and organized in colonies on F surfaces; on wrinkled surfaces, bacteria preferentially reside and locally orient along the direction of grooves. Larger *C. albicans* do not significantly respond the surface patterns, beyond a slight cell deformation along 1D patterns.

After inoculation and incubation, the cells on the surfaces were immobilized as described above thus providing stable surfaces for AFM and SEM imaging. Figure 3 summarizes the experimental results for the four strains or the four distinct surfaces, after an incubation time of 6 h. On flat PDMS surfaces (with residual roughness *R*_a_ ≈ 25 nm), the microbes are organized into a “colony” regime, characterized by cells stacked on different layers and extremely interconnected. This kind of disposition favors the formation of mature biofilms and is influenced by substrate surface energy and charge and nature of the bacterial cell membrane.^61−64^ On 1D wrinkled surfaces (1D), the bacterial species are ordered according to the pattern geometry in a single direction, whereas on 2D checkerboard (C) surfaces microorganisms were oriented in two different directions. On 2D herringbone (H) surfaces, an alignment similar to 1D is observed, even though the bacterial orientation follows the pattern structure. As expected, commensurate surface patterns induce specific bacterial arrangements, according to their geometry, confining bacteria into pattern depressions, and these are capable of reducing their interconnection. In order to generate multilayered aggregates and initiate colonization, bacteria must first fill the wrinkling valleys to be able to occupy the available surface. However, the quantitative interplay between additional surface area due to wrinkling, surface pattern tortuosity, and bacterial arrangement are not obvious and must be examined further. By contrast, the larger *C. albicans* do not appear to be significantly influenced by the surface patterns, beyond a modest deformation of individual cells along the 1D pattern direction.

**Figure 4:**
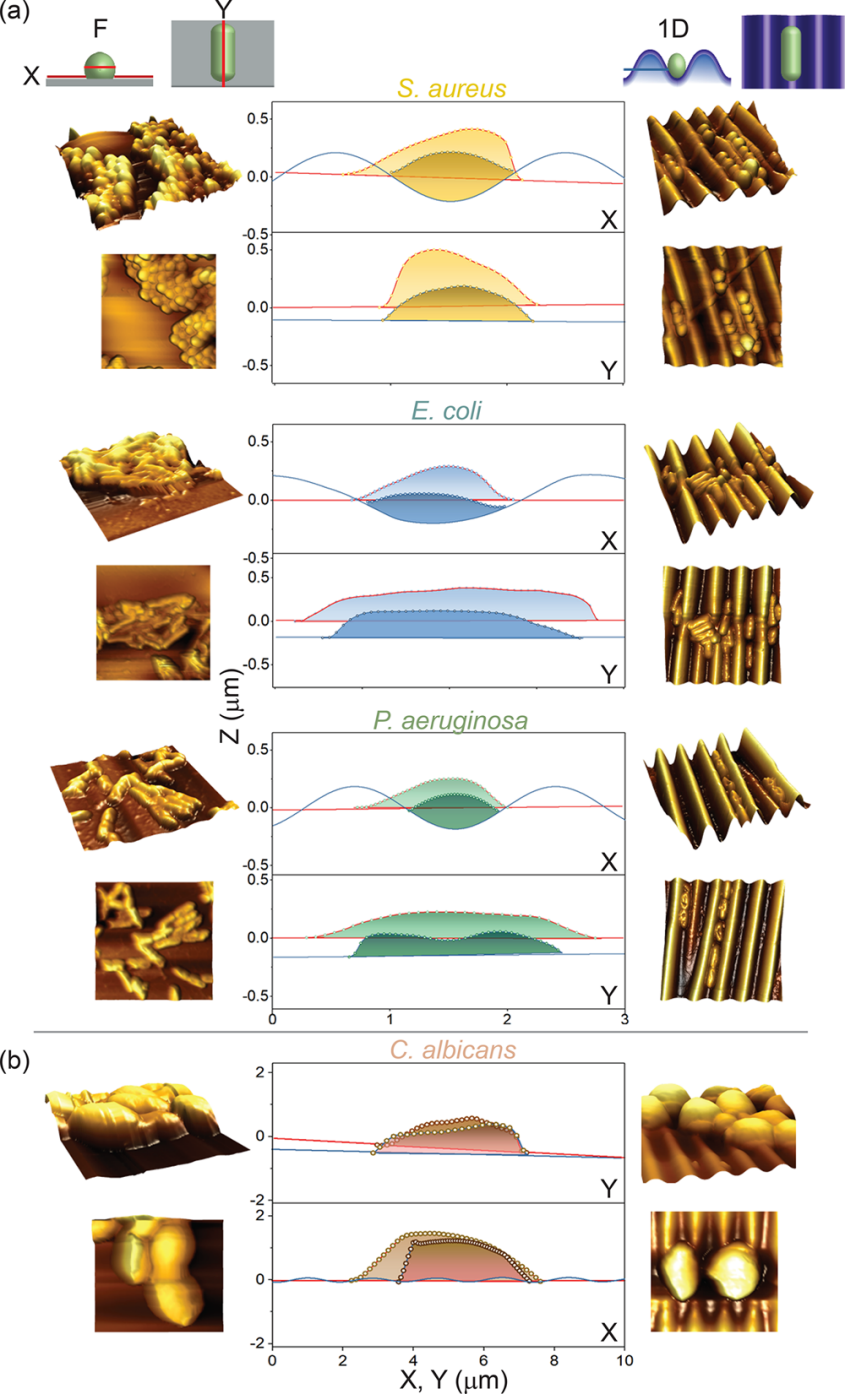
Top and side views of 3D AFM micrographs of (a) the different bacterial strains and (b) *C. albicans* on flat (left column) and 1D surfaces (right column). Line profiles extracted along the surface plane *x* and *y* directions. The *x* profile cuts through the cross-section of a single bacterial cell, whereas the *y* profile through the longitudinal section on a flat (red line) and a 1D wrinkled surface (blue).

By extracting the cross-sectional and longitudinal line profiles from the AFM images, it is possible to resolve the effect of curvature of the pattern valley on the microbes, compared to a flat surface, as shown in Figure 4. Bacteria exhibit clearly distinct organization as lumped and extensive aggregates on F surfaces (left), in contrast with the confined and groove-aligned arrangements in the 1D patterns (right). The *x* and *y* axis follow, respectively, the smaller and longer axis of the bacteria, while the red and blue traces indicate the F and 1D profiles. The spheroidal *S. aureus* attached onto a flat surface exhibits an average cell size (*x* and *y* cuts) of ≈1.4 μm. The 1D confinement reduces the average size (along X) to ≈1 μm. For rodlike bacteria *E. coli* K12 and *P. aeruginosa* on 1D surfaces, a similar effect is observed along the *y* cut, where the cell size is shaped by the confinement in the wrinkling valleys and a shrinkage is observed. By contrast, on F surfaces, the rodlike bacteria freely spread upon attachment, forming multilayered aggregates. *C. albicans* cells span over four wrinkling wavelengths, and their organization and size is on average not influenced by the surface topography, although a slight cross-sectional shrinkage and deformation is induced along the 1D pattern and appears to limit spreading and connectivity compared to F surfaces.

Table 2 summarizes the measurements of cell profiles along the cross-sectional and longitudinal direction of both F and 1D patterns averaged over 20 bacteria extracted from representative 20 × 20 μm^2^ AFM images. In order to account for intrinsic cell variability in size and shape, cross-sectional deformations were further estimated with over 200 cells for all the different strains and patterned surfaces, as reported in Figures S2 and S3. Furthermore, relatively large area and distinct area sampling is needed to mitigate the statistical effects of local heterogeneity and the microscale. The confinement and retention imposed by the wrinkling valleys is manifested on the bacteria, whose dimensions are commensurate with pattern wavelength and amplitude (λ = 2 μm, amplitude *A* = 0.2 μm), as observed previously on stainless-steel surfaces,^65^ of broad roughness spectrum, and in particular along grain boundaries. The fungus *C. albicans*, however, being double in size, can not be contained in the wrinkling valley, but upon adhesion, the cell membrane is also deformed by the pattern features as shown in Figure 4b.

**Table 2:** Microbial Cross-Sectional *a* and Longitudinal *b* Lengths Extracted from AFM Line Profiles on Flat and 1D Substrates from Figure 4

	*a*_F_ (μm)	*b*_F_ (μm)	*a*_1D_ (μm)	*b*_1D_ (μm)
*S. aureus*	1.2 ± 0.2	1.1 ± 0.2	0.9 ± 0.1	0.8 ± 0.1
*P. aeruginosa*	1.3 ± 0.3	3.4 ± 0.3	0.7 ± 0.1	2.5 ± 0.1
*E. coli* K12	1.1 ± 0.2	2.5 ± 0.2	0.8 ± 0.1	1.7 ± 0.1
*C. albicans*	4.2 ± 0.5	4.7 ± 0.5	3.9 ± 0.3	4.2 ± 0.3

### Microbial Orientation Maps on Patterned Surfaces

**Figure 5:**
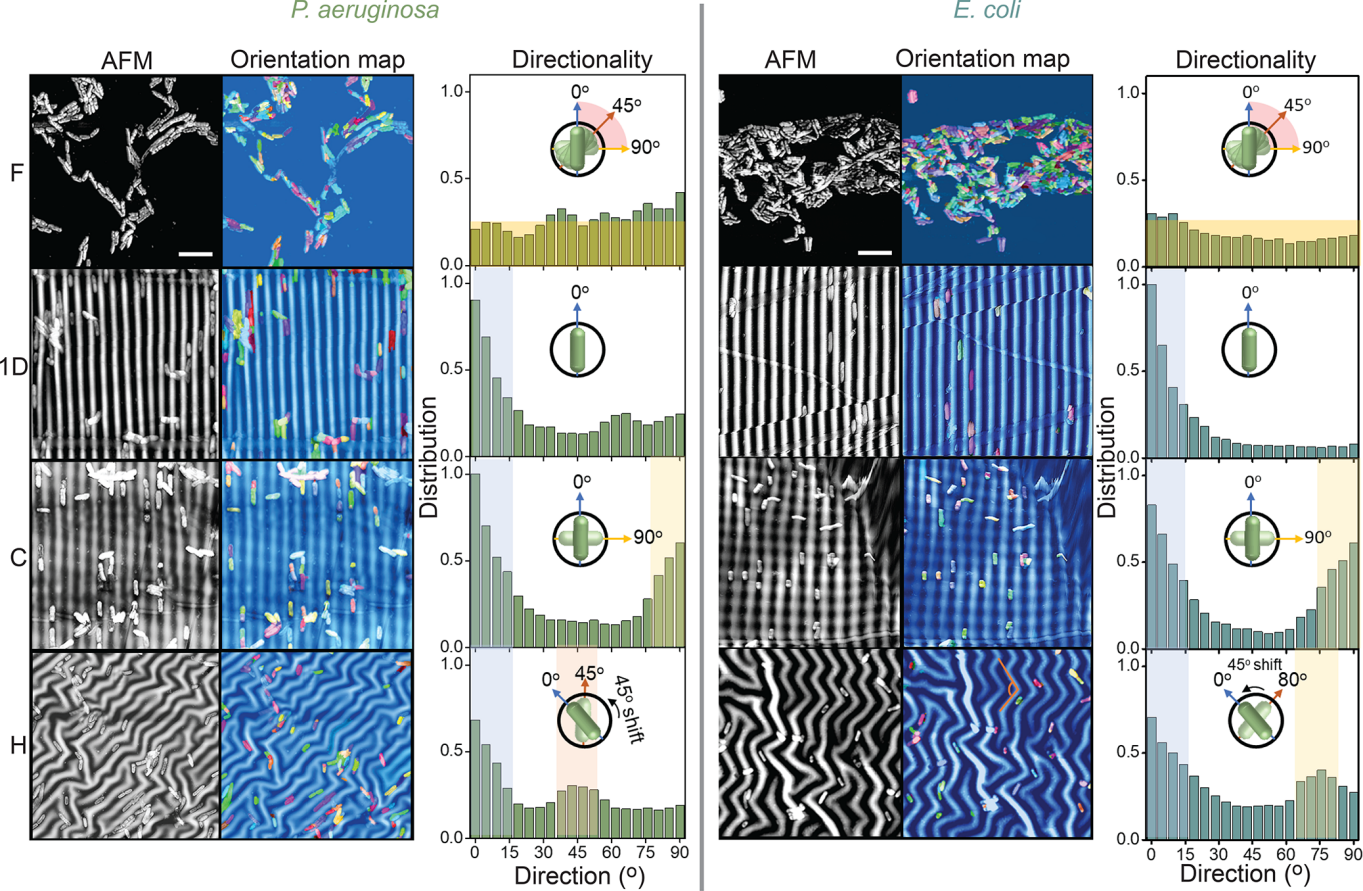
Orientational analysis for *P. aeruginosa* and *E. coli* K12 on F, 1D, C, and H surfaces. Left column: AFM images (50 × 50 μm^2^, acquired in tapping mode at 0.2 Hz). Middle column: image analysis (ImageJ using segmentation algorithm MorphLibJ) highlighting and isolating bacteria from the underlying surface, yielding a orientation color map via a local FFT and enabling the computation of orientation distributions (referenced to the N–S direction of the frame). Right column: azimuthal average of the overall FFT within 0–90°, normalized by the number of bacteria per frame (*N* ≈ 50). The scale bar represents 5 μm.

We next examine the degree of orientation imparted to the different microbial species by the model patterned surfaces. We first consider rodlike bacteria, *P. aeruginosa* and *E. coli* K12, whose orientation analysis is reported in Figure 5. Alignment is quantified in terms of the angular distributions of bacteria with respect to the prescribed directions imposed by the pattern geometry. AFM images (Figure 5a) are processed through a segmentation algorithm in ImageJ (Figure 5b), which imposes a shape descriptor (ellipses, in this case) and thresholds the shape, isolating the bacteria from the underlying surface. Once the shape boundary condition is defined, the remaining frame is accounted for as a constant and removed. To compute the orientation distribution, the segmented AFM image is divided into subimages where local FFT azimuthal averages are carried out in an angular range from 0 to 90°, with 0° defined as lying along the N–S direction of the frame. The average of the local FFTs is compiled as angular distribution in Figure 5c. As expected, bacteria on the F surfaces exhibit no preferential orientation. On the 1D surfaces, the distribution peaks at 0° aligned along the grooves, although a small fraction of *P. aeruginosa* also explore angles between 60 and 75°. For *E. coli* K12, however, the alignment to 0° is higher, which we associate with their smaller cross section, and thus greater constrain within the wrinkling valleys. On C surfaces, the bacteria are oriented along two well-defined angles set by the two generations of the orthogonal superposing pattern, i.e., along 0 and 90°. For *P. aeruginosa*, the distribution is weighted toward 0°, ascribed to the slightly higher local curvature of the second (vertical) generation wrinkling. Evidently, bacterial alignment is very sensitive to the local wrinkling amplitude. *E. coli* K12 cells are equally distributed along the two directions, corresponding to the matching amplitudes of the two wrinkling generations in this specimen. H surfaces are characterized by repetitive chevronlike domains, with an average angle of 90° associated with the simultaneous biaxial strain. Depending on the homogeneity of the applied strain, the kink angle can have a distribution, thus leading to a range of orientations. Provided that each pattern length is larger than the long axis of bacteria, H patterns can be construed as a sequence of local 1D patterns alternating in orientation. For this reason, the integration window of the azimuthal FFT average was shifted to −45° to 45° to align according to the herringbone main direction. For *P. aeruginosa*, the average kink was measured at 45°, and the bacterial cells were oriented accordingly. However, for *E. coli* K12, the average angle was 80°, much closer to the expected kink angle. The orientation mechanism appears analogous to that of 1D surfaces, whereby the bacteria follow the local director, and somewhat different for H surfaces, are characterized by an abrupt change in direction. While the elongated shape of rodlike bacteria can be expected to favor an orientation along their long axis, the impact of surface patterns on spheroidal bacteria, lacking intrinsic shape anisotropy, appears less clear.

**Figure 6:**
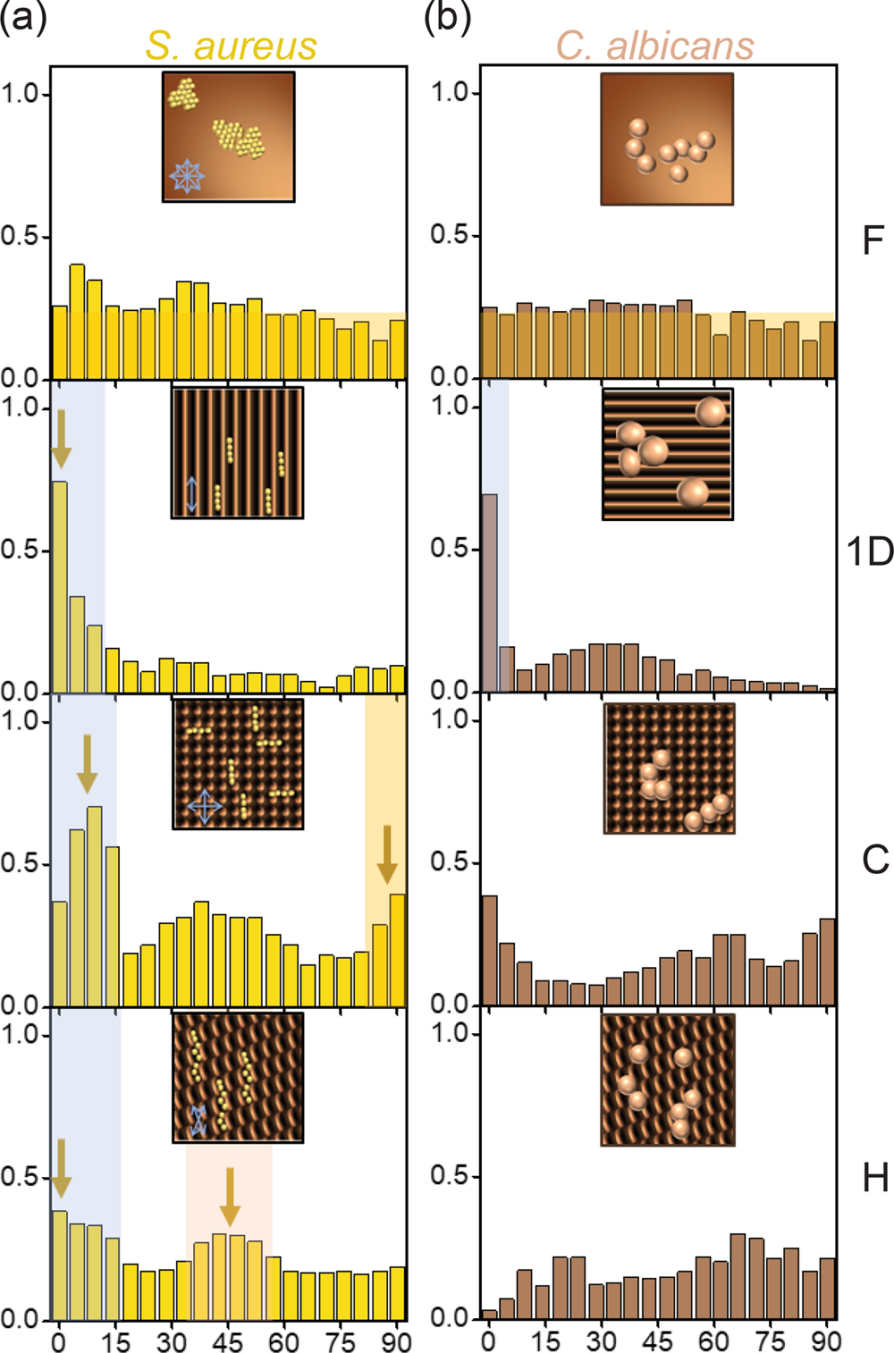
Orientational analysis for (a) spheroidal bacterium *S. aureus* and (b) fungus *C. albicans* on F, 1D, C, and H surfaces. *S. aureus* appears strongly oriented on the 1D surfaces and exhibits preferential orientations in both C (0 and 90°, and a broad intermediate band) and H (45 and 75°) surfaces. The larger *C. albicans* fungi exhibit no preferential orientation, except for the 1D pattern (albeit with lower statistics).

Orientation distributions for spheroidal *S. aureus* are reported in Figure 6a. The 1D patterns show the highest degree of alignment, with bacteria effectively confined within the wrinkling valleys (Figure 3). We note that 2D patterns are characterized by slightly lower curvatures compared to 1D surfaces, as a result of wave superposition between the two generations. This effect is more pronounced for C surfaces where the sequential wrinkling deviates from an exact sum of waves,^58,66^ associated with the fact that the second buckling event is excited onto a (previously) undulated surface. The resulting 2D pattern amplitude has been found to be reduced by a factor equal to the amplitude ratio *A*_2_/*A*_1_ of the two generations.^58,67^ Our data indicate that rodlike bacteria, with an intrinsic elongated shape, appear more efficiently constrained and directed by the wrinkling pattern, even when subjected to a lower amplitude. Spheroidal *S. aureus*, whose size is commensurate to the pattern scale, exhibits lower alignment on C surfaces, with a lower amplitude, compared to that on 1D patterns; therefore, additional correlation angles, beyond 0 and 90°, are observed. For H surfaces, the pattern amplitude is much closer to that of the 1D surface, and the pattern characteristic orientations are selected (0 and 45°) even though the number of cells aligned on average appears lower than that observed for rodlike bacteria.

For reference, the orientation maps for spheroidal *C. albicans*, with dimensions much larger than the patterned length scales, are shown in Figure 6b and generally show a lower degree of alignment. On 1D patterns, some alignment can be observed, although involving a statistically reduced number of cells, and thus with greater uncertainty. Overall, our data show that microbial alignment responds to specific features of surface pattern geometry, namely, in-plane correlations (in addition to amplitude and wavelength), and that cell dimension and aspect ratio are quantitatively reflected on their orientation distribution.

### Effect of Pattern Morphology on the Onset of Proliferation: *P. aeruginosa*

We next examine the spatiotemporal evolution of a selected bacterial strain, *P. aeruginosa*, based on AFM imaging of the four surface patterns F, 1D, H, and C. In order to define an objective surface area metric for comparison, one must consider that the undulations caused by wrinkling *increase* the area (3D), per unit of footprint area (2D). In practice, such an increase in surface area, on which bacteria can proliferate, could evidently be detrimental to bacterial proliferation, thus defeating the intended antimicrobial purpose. In experimental terms, an AFM scan area of *L*^2^ (referred to as the “footprint” or “projected” area) corresponds to the same *L*^2^ area for a flat surface, but a greater surface area for undulated surfaces, whose exact value depends on topography.

Considering a 1D sinusoidal profile, the excess length can be trivially estimated from strain as ϵ ≡ (*L*_*x*1_ – *L*_*x*0_)/*L*_*x*0_ and thus *L*_*x*1_ = (ϵ + 1)*L*_*x*0_. The undulated length for ϵ = 20%, for instance, should be 20% larger than its original length. Estimating the excess surface area is more complex, as stretching in the *x*-direction is accompanied by a compression in the *y*-direction. Since PDMS is incompressible (*V* ≡ *L*_*x*_*L*_*y*_*L*_*z*_ constant) and assuming constant thickness (*L*_*z*0_ ≃ *L*_*z*1_), the lateral compression could be estimated (as *L*_*y*1_ ≈ *L*_*x*0_/(ϵ + 1)), although is does not take into account the actual geometry of the strain field and the finite compression imposed by the stage clamps. We thus consider estimating the excess surface area by evaluating the line integral of a sine wave with amplitude *A* and wavelength λ, which can be experimentally measured, as
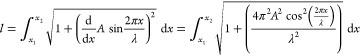
4where *x*_1_ and *x*_2_ are integration limits. Taking the measured *A* = 0.2 μm and λ = 2 μm (and reference AFM line scan of Δ*x* ≡ *x*_2_ – *x*_1_ = 20 μm), the perimeter of the sinusoidal pattern can be readily calculated and compared to the projected length, to yield  μm, and thus a surface area increase of  = 10%. However, since a sinusoidal profile is only strictly expected in the low deformation limit,^68^ we opt for a direct measurement of surface area based on the AFM data of the various surfaces, which we find to be reproducible and in broad agreement with geometric estimates.

We next consider that in the initial stages of proliferation the bacteria preferentially colonize depressions of surface pattern, such as grooves, valleys or wells.^31^ Taking the 1D sinusoidal pattern as illustration, one could expect that only approximately half (50%) of the pattern, i.e., the valleys, are effectively “available” to be occupied by bacteria. As a result, an “available” surface area to bacteria, *S*_A_, might thus be estimated by the product (surface area × its available fraction). Considering an increased area of 110% due to wrinkling and an available fraction of 50% valleys, for a 1D pattern, the available *S*_A_ is ∼55%. Experimentally, we obtain ≈67% of the overall surface (slightly greater than expected), rationalized in terms of the growing asymmetry of the sinusoidal profile toward higher deformations.^68^

**Figure 7:**
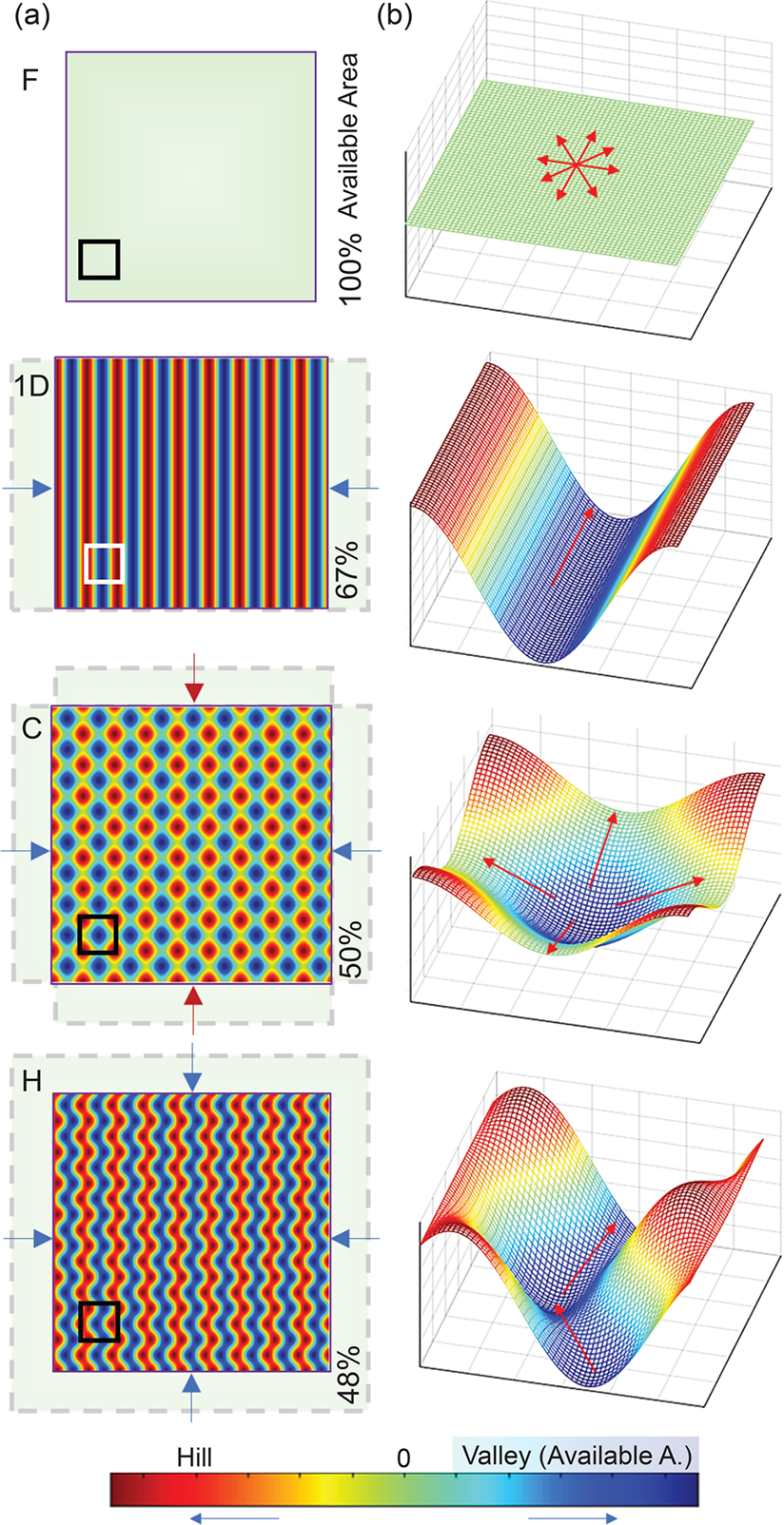
Estimation of available surface area *S*_A_ for F, 1D, C, and H topographies. (a) Calculated top view of all surfaces, indicating the excess surface area (dashed) due to strain; high and low amplitudes are shown, respectively in red and green, and the median (*z* = 0) in green. The available surface area *S*_A_ is shown in percent values. (b) 3D surface view depicting a magnified low amplitude areas (valley or well) with lateral dimension equal to λ, corresponding to the squares in (a).

A series of calculated F, 1D, C, and H patterns and experimentally measured available surface areas are reported in Figure 7a. These were estimated by thresholding the AFM images across the *z* = 0 plane and integrating the area fraction of negative amplitude (Figure S4). The green color indicates *z* ∼ 0, and the variation in amplitude is defined as positive (hills, shown in red) and negative (valleys, in blue). For the reference F surface, 100% of the area is considered available. The C and H surfaces display *S*_A_ of ≈50 and ≈48%, respectively, associated with their increased topographical complexity. The sequential wave superposition in C surfaces generates an array of “pits” and “dimples”, and *S*_A_ is reduced by depressions that bacteria must overcome along the *z*-axis; the simultaneous wave interference in H surfaces generates zigzag patterns, which bacteria must negotiate within the *xy*-plane.

**Figure 8:**
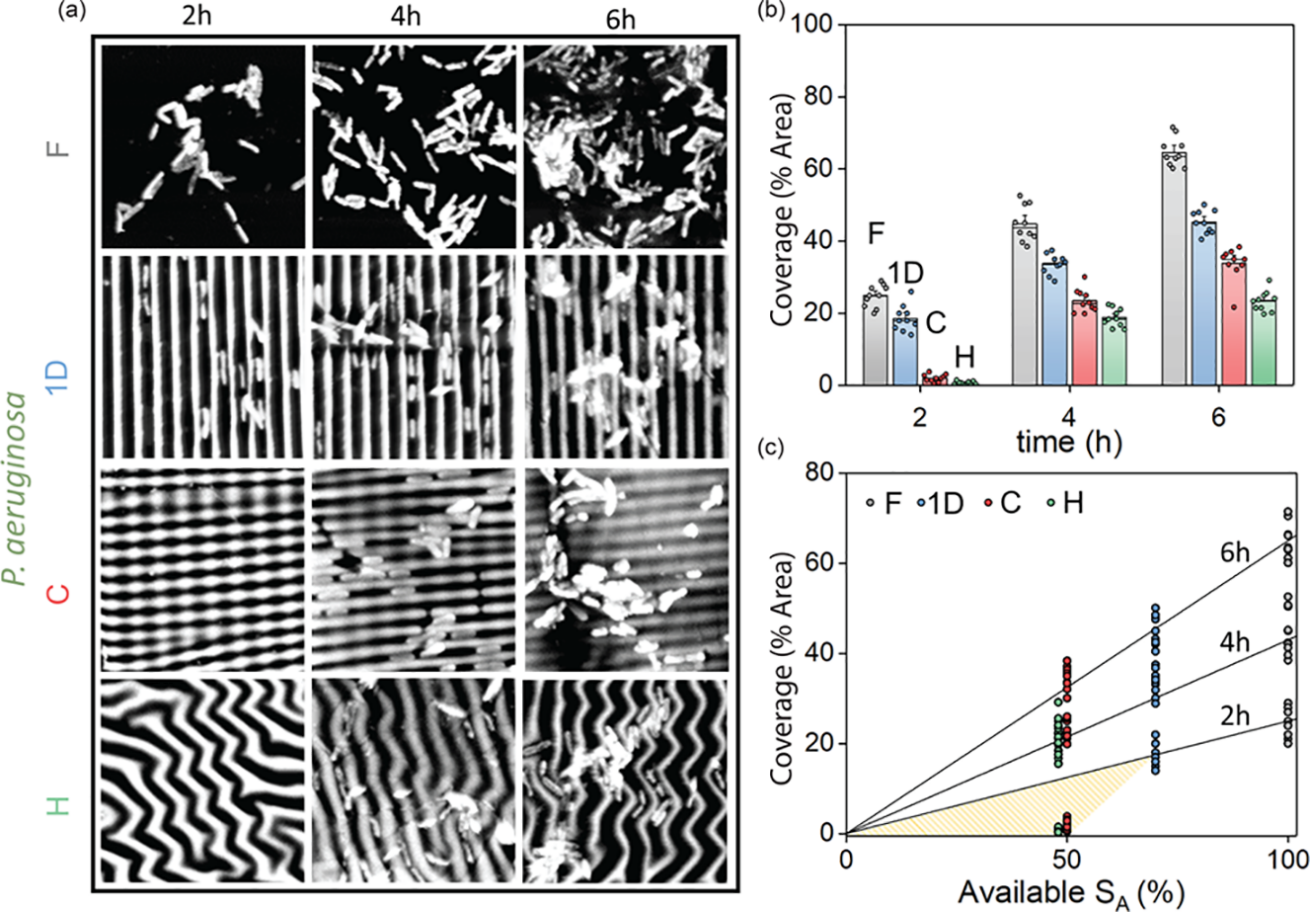
Coverage analysis for *P. aeruginosa*. (a) AFM micrographs (20 × 20 μm^2^, tapping mode, 0.2 Hz) of the bacteria sampled at 2, 4, and 6 h, over the different wrinkled substrates. (b) Calculated coverage data reported by overlapped scatter and bar plots extracted from the experimental AFM micrographs. Coverage is calculated as the percentage area occupied by bacteria, averaged over a series of three 100 × 100 μm^2^ micrographs acquired in different location of the specimen, and for 3 different samples. (c) Bacteria areal coverage as a function of the available surface area (pattern depressions). The black solid lines represent the linear correlation between the available surface area for a flat surface (100%) and the experimental areal coverage. Pattern formation reduces the available surface area and the areal coverage decreases accordingly at 4 and 6 h. At 2 h, the 2D surfaces deviates form linearity meaning that the additional reduction in coverage at the onset of proliferation is not only due to a reduction in surface area but also to the specific pattern topography.

Proliferation was quantified in terms of the areal coverage of *P. aeruginosa* by AFM, at prescribed incubation times (0, 2, 4, and 6 h), collecting population statistics on three separate specimens of the same surface type, and three distinct surface locations (100 × 100 μm^2^) of each of the different wrinkled topographies (Figure S5). Furthermore, to quantify proliferation over larger and distinct sample areas, optical microscopy was also carried out (300 × 200 μm^2^ images, three sample locations, provided in Figures S6–S9). Coverage was estimated by using the same method reported in Figure 5 to build the orientation maps. Specifically, the underlying surface pattern was subtracted and the area of the ellipsoidal objects (bacteria) was normalized by the projected area of the image, obtaining the bacteria areal coverage (%). The results are reported in Figure 8 where Figure 8a shows 20 × 20 μm^2^ AFM details of the temporal evolution of *P. aeruginosa* over the wrinkled topographies and Figure 8b shows the overlapped scatter and bar plots of the coverage data over time. On F surfaces (native PDMS nanometer roughness), *P. aeruginosa* are randomly distributed and progressively aggregate into multilayered lumps, indicative of colony behavior, leading to biofilm development, favoring interconnections and strengthening adhesion. This coverage appears linear in time. For comparison, the same coverage analysis was carried out on the optical microscopy data and the results, in line with those obtained by AFM, are reported in Figure S10. In order to account for the statistical sample variation and local heterogeneity within samples, coverage was independently calculated (as the percentage area occupied by bacteria) at three or four distinct locations per specimen for three specimens and subsequently averaged as shown in Figure 8b, obtained from AFM, and in Figure S10, obtained from complementary optical microscopy analysis. In general, AFM imaging enables a more precise thresholding, and the data are thus selected for further analysis.

While the water contact angles, respectively, 106, 105, 98, and 85° for F, 1D, H, and C, vary somewhat for the patterned surfaces, we believe these modest changes cannot account for the experimental observations. These contact angles are considerably lower than those characteristic of superhydrophobic surfaces (∼150°) achieved in certain high aspect ratio hierarchical wrinkled surfaces, and resulting in Cassie–Baxter behavior;^23,27^ the patterns considered in this work thus yield Wenzel behavior, as bacteria are generally found to reside in pattern depressions. Furthermore, the trend between areal coverage and contact angle is nonmonotonic, being reversed for H and C surfaces; finally, the vanishingly small difference (∼1°) in contact angle between F and 1D results in a significant change in coverage.

Instead, wrinkling appears to impact bacterial spreading in other ways; the reduction in “available” surface area *S*_A_ appears to displace bacteria toward valleys, hence, ordering them according to predetermined pathways. In turn, the specific topography can exert mechanical frustration that impacts the onset of colony behavior. On 1D surfaces, pattern depressions appear to be able to contain bacteria within valleys at 2 h; however, the accumulation of bacteria in valleys appears to effectively “even out” the surface perceived by subsequently approaching cells, which can freely adhere with no topographical constrains. The overall coverage is nonetheless lower than that for F surfaces at all times. By contrast, both C and H patterns (biaxial) nontrivially affect the onset of bacterial proliferation, with only residual coverage observed at 2 h and significantly reduced coverage thereafter.

In order to rationalize the coverage data in terms of the “available” surface area arguments above, Figure 8c reports the experimental bacteria coverage as a function of *S*_A_. The data collapse, within measurement uncertainty, of the coverage results for distinct surfaces, at the *same* time demonstrates the merit of the *S*_A_ estimates detailed above. In a first approximation, coverage thus appears linearly proportional to *S*_A_, and C and H surfaces offer considerable benefits compared to 1D (and F) surfaces, which we associate with their tortuosity. Moreover, we observe a significant deviation for C and H data at the early stages of proliferation (up to 2 h): The data deviate from linearity, and the onset of proliferation appears to be effectively suppressed, or delayed by a few hours, on these 2D topographies. For smaller pattern dimensions with respect to microbial dimensions, an analysis in terms of specific contact points of attachment is often employed.^46^ In the present case, pattern curvatures are commensurate with those of bacteria, and pattern amplitudes are smaller than microbial dimensions. As such, the accessible surface area analysis appears appropriate. Finally, while proliferation is influenced by *S*_A_, its onset is additionally impacted by specific, local pattern topography which likely frustrates the bacterial spatial arrangement, connectivity, and motility, hindering the formation of multilayered aggregates.

## Conclusions

In this paper, we experimentally examine the effect of microscale surface patterning on microbial attachment and proliferation. Significantly, we investigate surfaces with comparable roughness, in order to decouple specific effects of topography on model bacteria and a fungus. In addition to a flat (F) control surface (*R*_a_ ≃ 0.02 μm), we selected uniaxial sinusoidal (1D), biaxial herringbone (H), and checkerboard (C) with λ ≃ 2 μm and *A* ≃ 2 μm and similar roughness, *R*_a_ ≃ 0.07 μm. We select three model bacterial strains, namely, *S. aureus*, *P. aeruginosa*, and *E. coli* K12, and one fungus, *C albicans*, of various characteristic shape (spheroidal or ellipsoidal), Gram stain, and size. Our experimental results establish that bacteria commensurate with surface pattern length scales align closely with the local pattern directors and, at early stages, within valleys or depressions in the topography. As expected, incommensurate *C albicans* is comparatively less affected by the surface patterns, beyond a modest alignment at individual cell level.

Bacterial proliferation, specifically of *P. aeruginosa*, reveals a significant response to surface topography whereby coverage is reduced in the order F > 1D > C > H. This mitigation is rationalized in terms of an “available” surface area *S*_A_ to bacteria, which takes into account both the *increase* in surface area due to undulations and a reduction of area fraction associated with the pattern depressions, i.e., below the median plane. Experimental data for bacterial coverage is effectively reduced into a master curve, as a function of *S*_A_. However, we find that C and H topographies impose an additional constraint to the onset of proliferation, effectively delaying it by several hours, during which there is only residual bacterial surface coverage (up to 6 h examined in this work). We associate this frustration to the tortuosity of C and H patterns, in either the *z*- or *xy*-planes, imposing prescribed relative orientational changes commensurate with bacterial dimensions and thereby hindering their proliferation.

Overall, we believe that this method of exploiting surface wrinkling instabilities of bilayer soft materials (or surface replicas) provides a straightforward and economical route to patterning large areas of surfaces with consequential antimicrobial action. We show that roughness metrics alone are not sufficient to describe surface topography and that pattern tortuosity plays a significant role in microbial attachment and proliferation. Multiaxial patterning, either by simultaneous or sequential application of strain, is shown to provide a facile means to augmenting antimicrobial action. Evidently, our study also opens many lines of enquiry, regarding (i) the quantitative impacts of pattern λ and *A*, (ii) the role of surface chemistry and surface energy, including its differential impact in Gram positive or negative bacteria, and the generality of the findings, (iii) the possible role of detailed topographic profiles (such as higher surface modes attained upon increasing strain or with hierarchical generations), as well as in-plane features, such as the segment zigzag length of checkerboard (C) surfaces, (iv) the continuum toward nanoscale patterning, whose mode(s) of action differs from those at the microscale, and (v) the development of a robust and simple surface metric, or a small set of metrics, with predictive ability for antimicrobial action.

## Supporting Information Available

Water contact angle measurement for the different patterned and flat PDMS surfaces, microbes cross-sectional deformation for the different wrinkled surfaces, counting of *S. aureus* on 1D surfaces, *P. aeruginosa* AFM coverage data, available surface area of attachment of wrinkled topographies, optical microscopy coverage images for *P. aeruginosa* on F surfaces, optical microscopy coverage images for *P. aeruginosa* on 1D surfaces, optical microscopy coverage images for *P. aeruginosa* on C surfaces, optical microscopy coverage images for *P. aeruginosa* on H surfaces, coverage analysis for *P. aeruginosa* on the different surfaces (PDF)
