# Supplementary material for: Microbial Response to Micrometer-Scale Multiaxial Wrinkled Surfaces
Source: ACS Appl Mater Interfaces. 2022 Jun 14;14(27):31463–73. doi: 10.1021/acsami.2c08768 (PMC9284519; doi:10.1021/acsami.2c08768)
Supplement: Supplementary file 1 — am2c08768_si_001.pdf [file am2c08768_si_001.pdf]

Supporting Information
Microbial response to micronscale multi-axial wrinkled surfaces

Luca Pellegrino, Lukas Simon Kriem, Eric S. J. Robles and João T. Cabral*

Department of Chemical Engineering, Imperial College London, London SW7 2AZ, U.K.

Fraunhofer Institute for Interfacial Engineering and Biotechnology IGB, Nobelstrasse 12, 70569 Stuttgart, Germany

Procter & Gamble, Newcastle Innovation Centre, Newcastle upon Tyne NE12 9TS, U.K.

E-mail: j.cabral@imperial.ac.uk

1 Water contact angle of the patterned surfaces F, 1D, C and H

Patterned surfaces were fabricated following procedures reported previously.[1–4] In short, single wrinkling (1D) or sequential wrinkling wave superposition (C, H) was carried out to fabricate model surfaces of similar roughness but distinct topography, as described in the main paper. The surfaces were then replicated onto flat PDMS to eliminate effects related to the plasma oxidation procedure, enabling a direct comparison with flat (F) PDMS surfaces. Water contact angle measurements reveal that the 2D samples exhibit a slightly lower contact angle, namely 85° for C and 98° for H surfaces, compared to 1D (105°) and flat F (106°) surfaces. Since the patterned, plasma-exposed, samples were replicated into virgin PDMS, these modest changes can be ascribed solely to pattern topography, since the surface chemistry is identical.

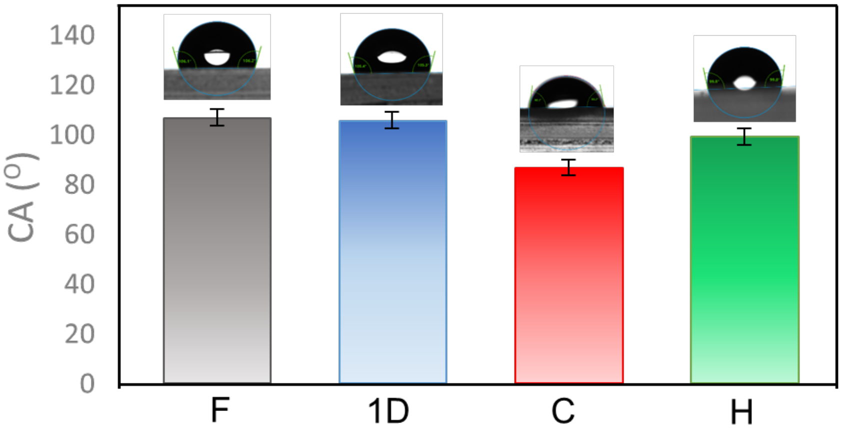

Figure S1 Water contact angle measured for the different F, 1D, C, and H PDMS surfaces reported in the main paper.

2 Estimate of microorganisms cross-sectional deformation on wrinkled surfaces

To assess the mechanical deformation exerted by the sinusoidal wrinkles, line profiles extracted from AFM images (Fig. 4 main paper) were further examined to compute their the cross-sectional deformation. The cross-sectional deformation was calculated as $D_{cs} = (d_l - d_t)/(d_l + d_t)$, where d_l and d_t are respectively the horizontal and vertical cross-sectional diameters. When confined to the recessed regions of the sinusoidal valley, microorganisms adapt their shapes to accommodate the mechanical constrain induced by the pattern. Overall, all bacterial strains showed a consistent deformation 0.5 and 0.8, with the highest deformation registered for *E. coli* on 1D surfaces. *C. albicans* is not significantly affected by the wrinkled topographies due to the larger size (2-3 times the wrinkling wavelength), although a higher deformation is observed for 1D patterns.

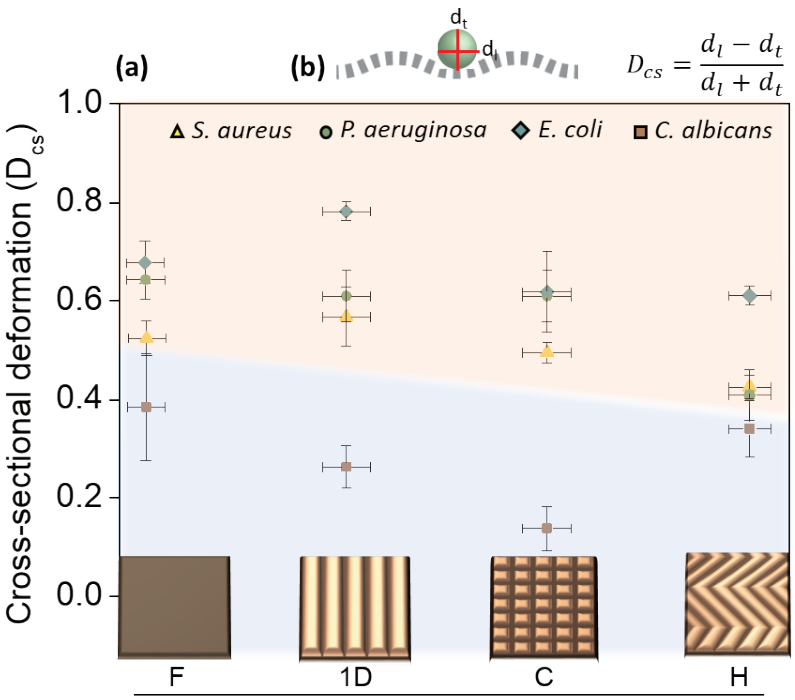

Figure S2 Microbial cross sectional deformation on wrinkled surfaces, calculated extracting the cross-sectional diameters d_t and d_l from the AFM line profiles.

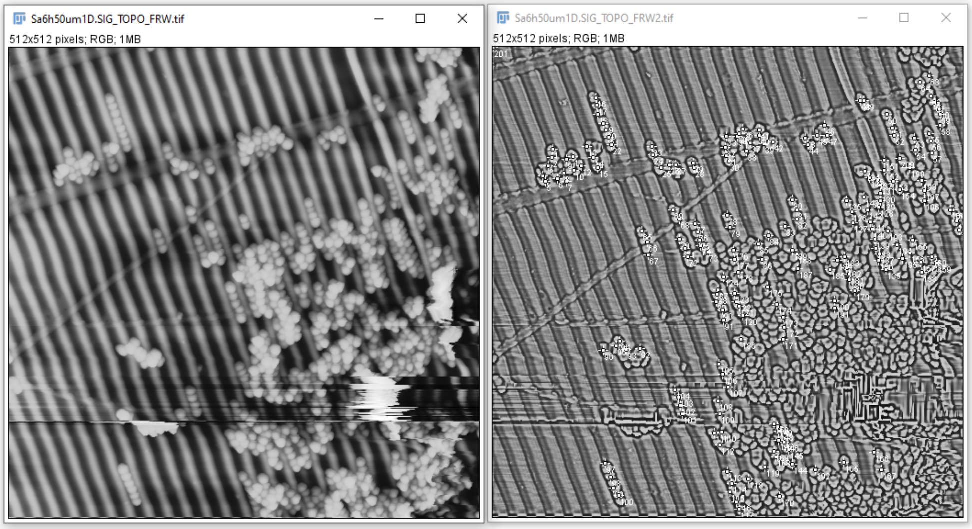

Figure S3 Illustration of bacterial cell counting from 100×100 μm^2 AFM imaging of a 1D wrinkling surface seeded with *S. aureus* after 6h. The image on the right was filtered and thresholded to enable the deformation of bacteria located in patter valleys to be extracted.

3 Available surface area of attachment of wrinkled topographies

As discussed in the main text, in Fig. 7, surface patterning increases the overall surface area, although the generation of specific three-dimensional topographies arises into protruding (hills) and recessed (valley) regions, characterising respectively the positive and negative amplitude of the sinusoidal pattern. Bacteria preferentially adhere in the valley region of the surface, maximising surface contact area, and therefore pattern formation inherently reduces the available surface area of attachment. To compute the surface area in the valley regions, the experimental AFM images were analysed by thresholding the positive (white) from the negative (black) amplitude areas of each pattern. The actual valley area was then computed by measuring the pixel area density. For a flat surface, the entire surface is available for attachment (100%). A 1D pattern reduces the available surface area to 65%, whereas the C and H (2D) surfaces respectively display 50% and 48% of available area. The different topographical order of the 2D surfaces, alternating negative and positive areas, and the density and typology of the repeat pattern (squares, triangles) further reduces the effective available area.

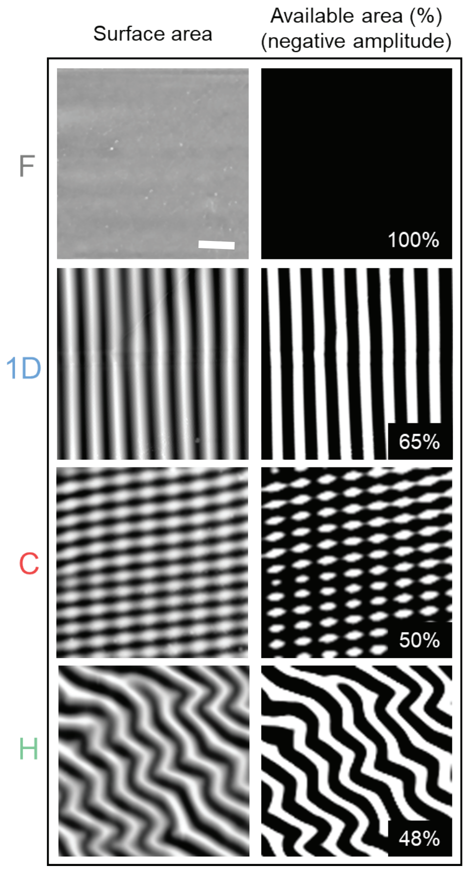

Figure S4 Available surface area S_A of attachment for flat surface (F) and wrinkled topographies (1D, C, H). Values extracted from experimental AFM data by thresholding the negative amplitude areas (black) and computing the corresponding pixel area fraction (using ImageJ).

4 Surface coverage data for *P. aeruginosa* estimated by AFM and OM imaging

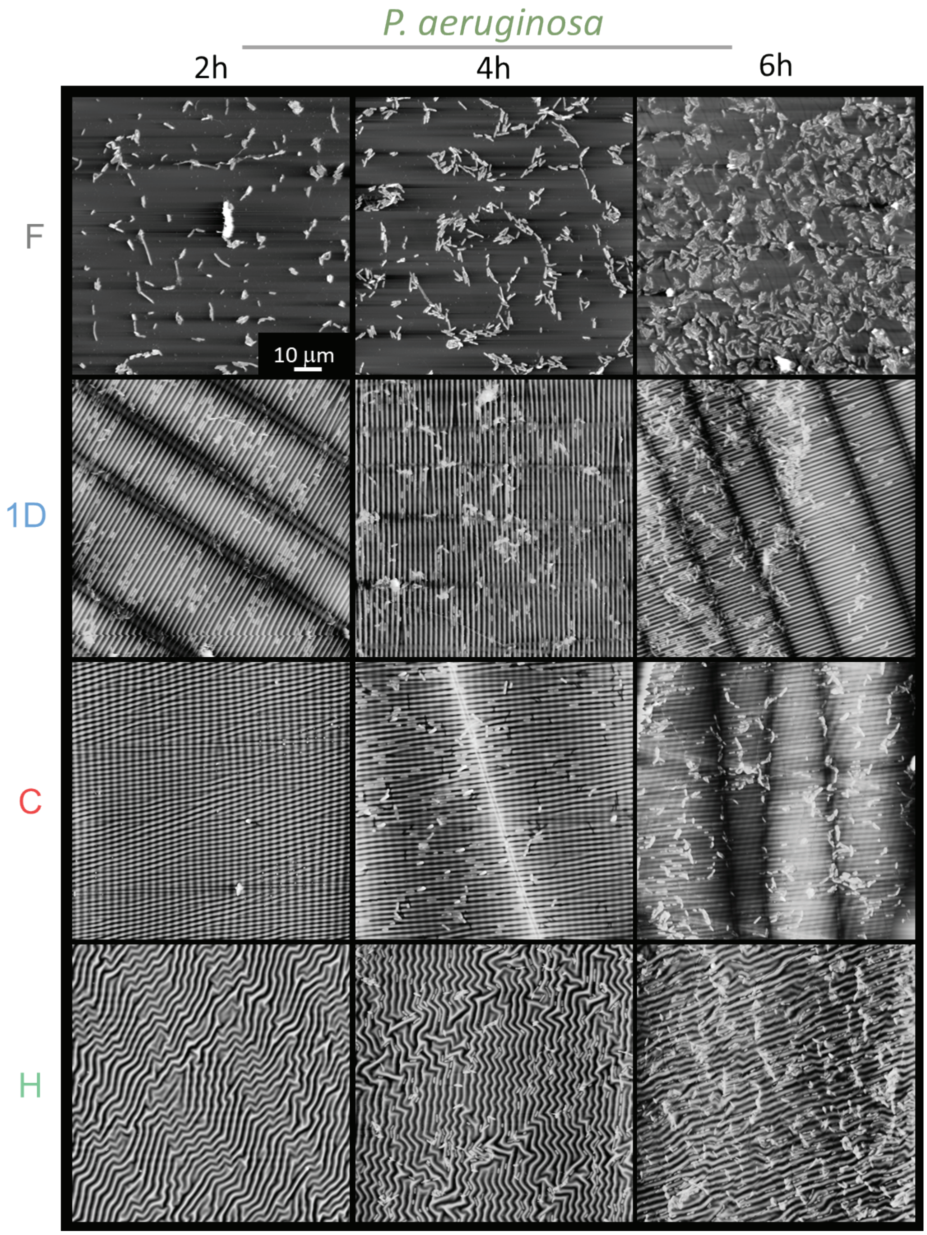

Figure S5 AFM images (100×100 μm^2) employed for the coverage analysis for *P. aeruginosa* compiled in Fig. 8 of the main paper.

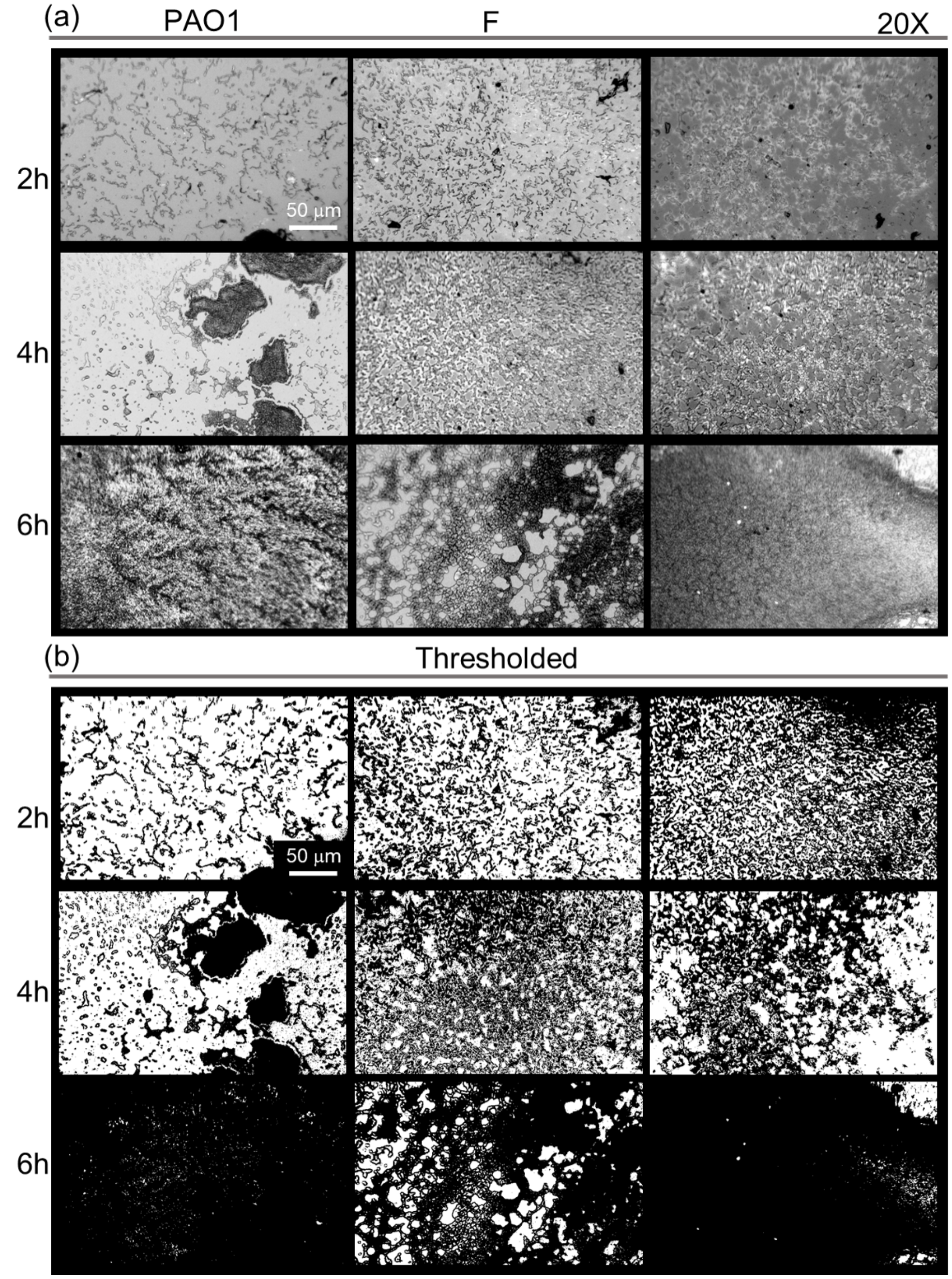

Figure S6 Optical microscopy (OM) images (300×200 μm^2) employed for the coverage analysis for *P. aeruginosa* on F surfaces, measured at three distinct locations of the same sample. Panel (a) shows the unprocessed image and (b) is the thresholded image, highlighting the bacterial coverage, shown in black.

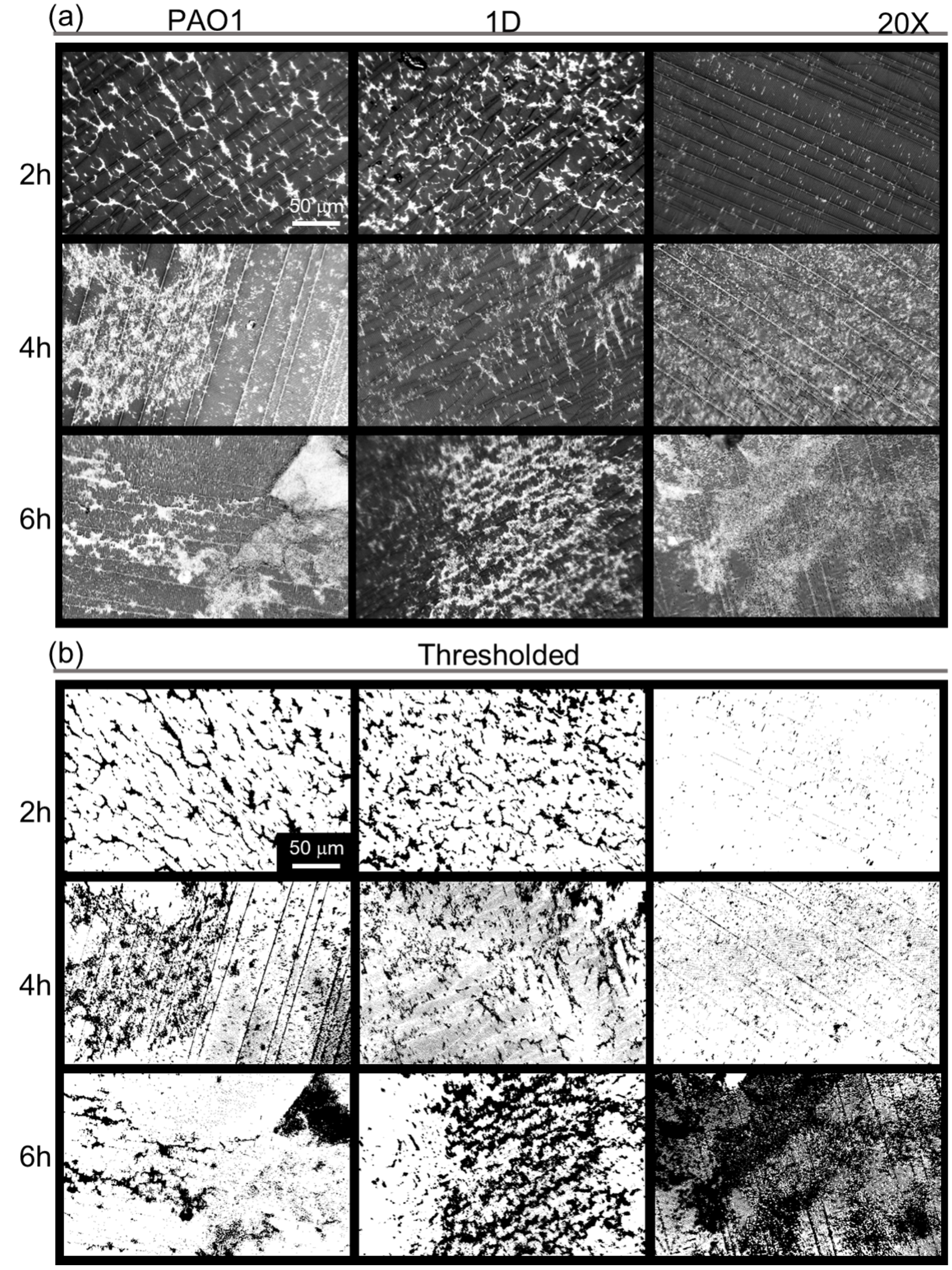

Figure S7 Optical microscopy (OM) images (300×200 μm^2) employed for the coverage analysis for *P. aeruginosa* on 1D surfaces, measured at three distinct locations of the same sample. Panel (a) shows the unprocessed image and (b) is the thresholded image, highlighting the bacterial coverage, shown in black.

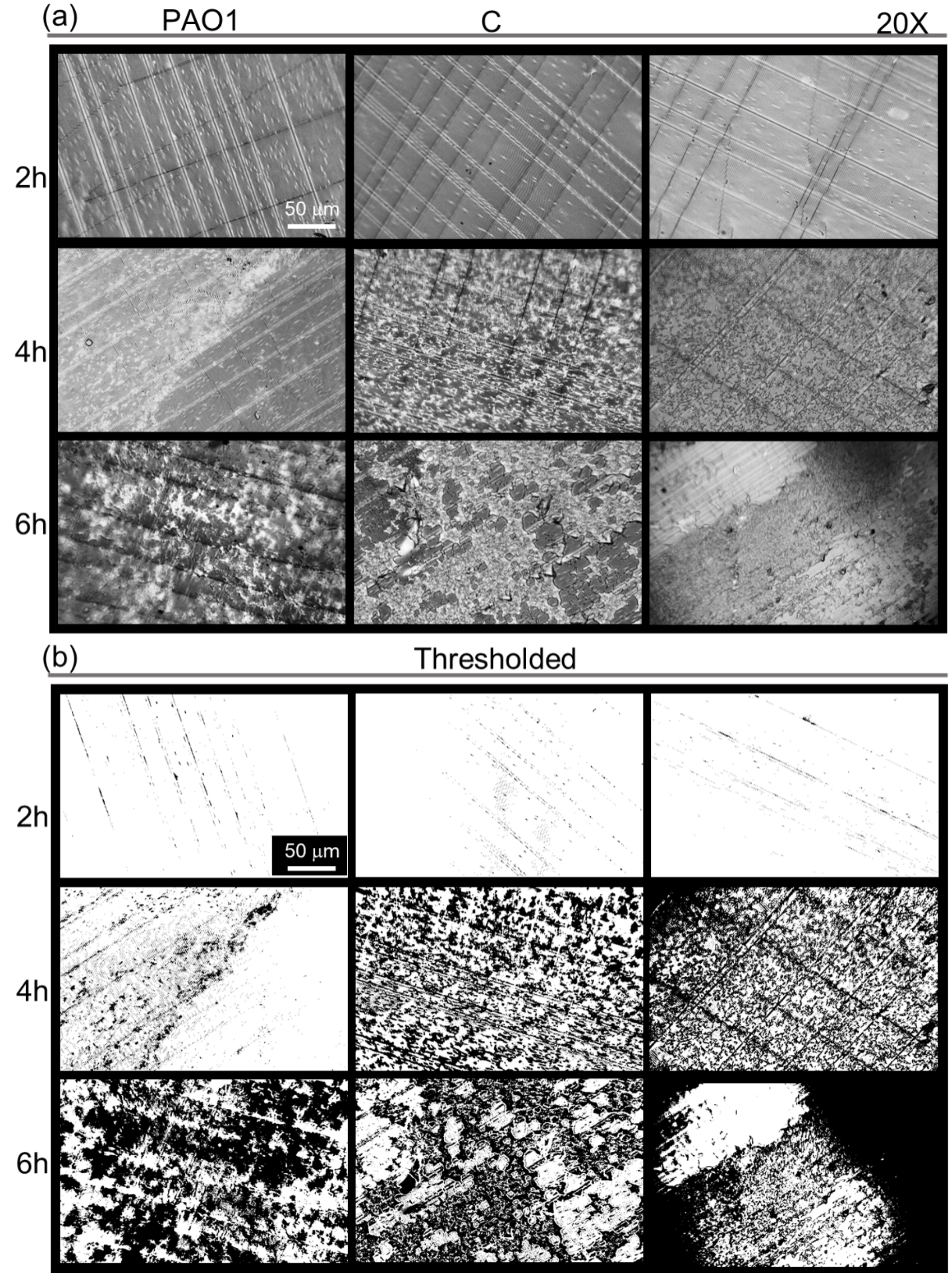

Figure S8 Optical microscopy (OM) images (300×200 μm^2) employed for the coverage analysis for *P. aeruginosa* on C surfaces, measured at three distinct locations of the same sample. Panel (a) shows the unprocessed image and (b) is the thresholded image, highlighting the bacterial coverage, shown in black.

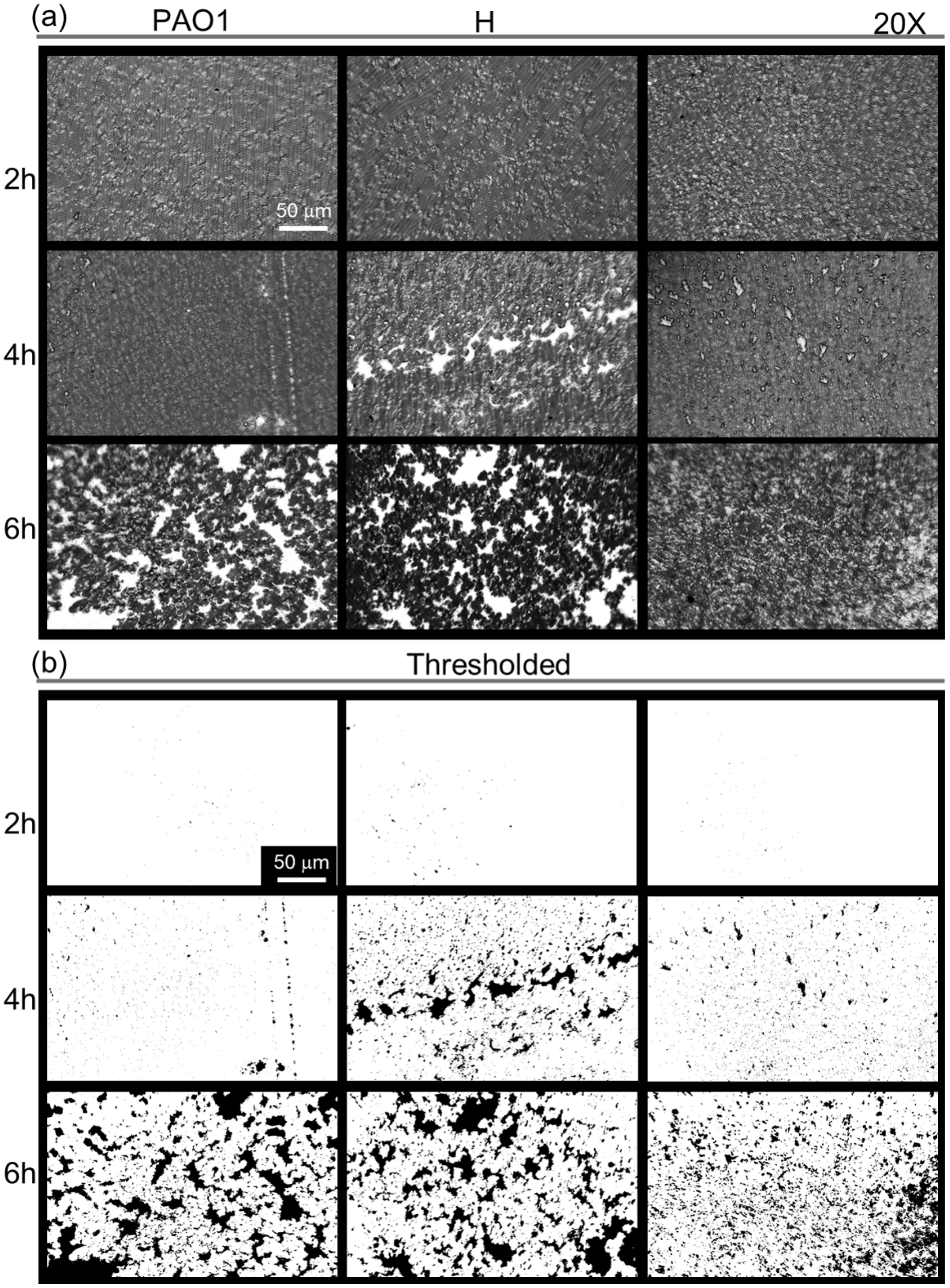

Figure S9 Optical microscopy (OM) images (300×200 μm^2) employed for the coverage analysis for *P. aeruginosa* on H surfaces, measured at three distinct locations of the same sample. Panel (a) shows the unprocessed image and (b) is the thresholded image, highlighting the bacterial coverage, shown in black.

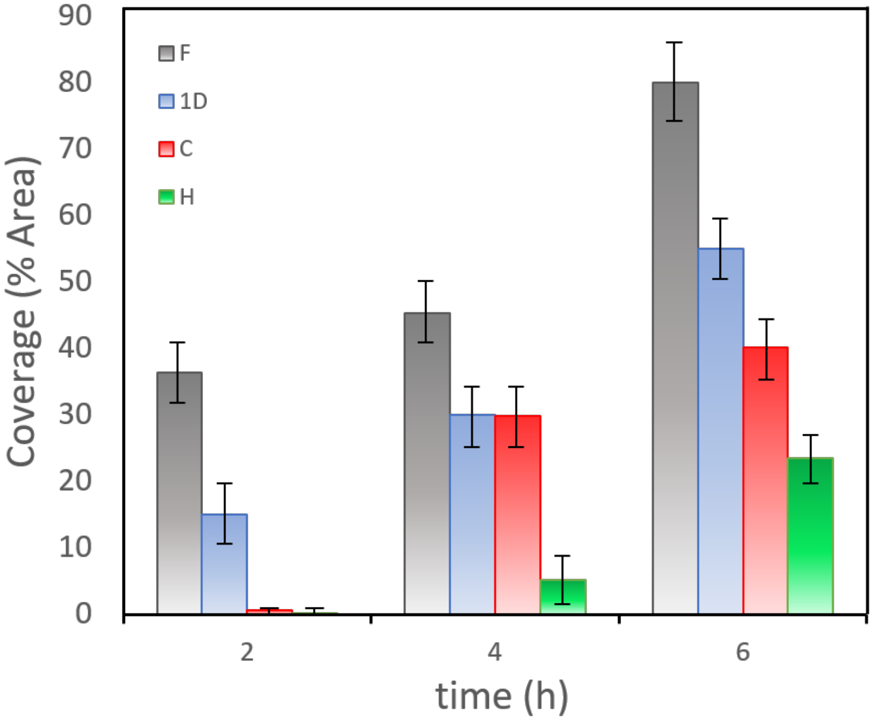

Figure S10 Bacterial coverage analysis for *P. aeruginosa* based on optical microscopy (OM) data reported in Fig. S6, Fig. S7, Fig. S8, Fig. S9. The surface coverage values obtained by OM are generally (10%) higher than those computed from AFM imaging (Fig. 8 of the main paper) and also exhibit larger uncertainties. The overall conclusions and ranking of the surface patterns in terms of their bacterial impact is unchanged. AFM images permit a more precise thresholding and thus estimation of bacterial coverage, albeit being more susceptible to local heterogeneity and sampling issues. The combination of AFM and OM estimates provides complementary and robust estimates of micro and macroscale behaviour.

Notes and references

[1] Chiche, A.; Stafford, C. M.; Cabral, J. T. Complex micropatterning of periodic structures on elastomeric surfaces. *Soft Matter* **2008**, *4*, 2360–2364

[2] Bayley, F. A.; Liao, J. L.; Stavrinou, P. N.; Chiche, A.; Cabral, J. T. Wavefront kinetics of plasma oxidation of polydimethylsiloxane: limits for sub-μm wrinkling. *Soft Matter* **2014**, *10*, 1155–1166

[3] Pellegrino, L.; Khodaparast, S.; Cabral, J. T. Orthogonal wave superposition of wrinkled, plasma-oxidised, polydimethylsiloxane surfaces. *Soft Matter* **2020**, *16*, 595–603

[4] Pellegrino, L.; Tan, A.; Cabral, J. T. Ripple Patterns Spontaneously Emerge through Sequential Wrinkling Interference in Polymer Bilayers. *Physical Review Letters* **2022**, *128*, 058001
